# A Comparative Study of Ten Pear (*Pyrus communis* L.) Cultivars in Relation to the Content of Sugars, Organic Acids, and Polyphenol Compounds

**DOI:** 10.3390/foods11193031

**Published:** 2022-09-30

**Authors:** Asima Akagić, Amila Oras, Fuad Gaši, Mekjell Meland, Pakeza Drkenda, Senad Memić, Nermina Spaho, Sanja Oručević Žuljević, Igor Jerković, Osman Musić, Metka Hudina

**Affiliations:** 1 Faculty of Agriculture and Food Sciences, University of Sarajevo, Zmaja od Bosne 8, 71 000 Sarajevo, Bosnia and Herzegovina; 2 Department of Horticulture, Norwegian Institute of Bioeconomy Research, NIBIO Ullensvang, Ullensvangvegen 1005, NO-5781 Lofthus, Norway; 3 SBH d.o.o. Sarajevo, Stari Drum 159, 71 210 Ilidža, Bosnia and Herzegovina; 4 Faculty of Chemistry and Technology, University of Split, Ulica Ruđera Boškovića 31, 21000 Split, Croatia; 5 Department of Agronomy, Biotechnical Faculty, University of Ljubljana, Jamnikarjeva 101, 1000 Ljubljana, Slovenia

**Keywords:** primary metabolites, secondary metabolites, sustainable traditional genotypes, pulp, skin

## Abstract

Traditional pear cultivars are increasingly in demand by consumers because of their excellent taste, the possibility of use in sustainable food production systems, convenience as raw materials for obtaining products of high nutritional quality, and perceived health benefits. In this study, individual sugars, organic acids, and polyphenols in the fruits of nine traditional and one commercial pear cultivar during two growing seasons were determined by HPLC. A significant influence of cultivars, growing years, and their interaction on the content of analyzed primary and secondary metabolites was determined. The commercial pear cultivar ‘Président Drouard’ and traditional cultivars ‘Dolokrahan’, ‘Budaljača’, and ‘Krakača’ had a lower content of all analyzed sugars. Overall, traditional pear cultivars had higher total polyphenols in the peel and pulp than ‘Président Drouard’, with the exception ‘Takiša’ and ‘Ahmetova’. High polyphenol content detected in ‘Budaljača’, ‘Dolokrahan’, and ‘Krakača’ shows the utilization value of traditional pear germplasm. The obtained data can serve as practical supporting data for the use of traditional pears in the neutraceutical, pharmaceutical, and food industries.

## 1. Introduction

Pear (*Pyrus* spp.) has a special place in the agricultural production of Bosnia and Herzegovina, as it can be grown in a wide range of climatic and soil conditions, has a prolonged maturation from early summer to late autumn, and produces fruit with good nutritional value, which is classified as nutrient-dense [1]. Pear fruits are an excellent source of dietary fiber; amino acids; minerals such as sodium, potassium, calcium, magnesium, and iron; and vitamins, which are very important health-beneficial biocomponents [2]. A major factor that determines consumer preferences among health-conscious people is the general composition and, in particular, the antioxidant properties of the fruit. The most important components of pear fruits are sugars, phenol compounds (PC), and organic acids. Their composition and concentration strongly influence taste and aroma, as well as the sensory impression in general. Aside from the sensory experience, these compounds are important for the nutritive characteristics of the fruit and its shelf life. An analysis of individual sugars and organic acids is a useful tool for determining fruit products’ authenticity. These compounds play a notable role in the formation of gel and its consistency in gelatinous products and also in the production of juices and nectars since the sweetness index (SI) is defined according to their concentrations. In addition, sugars play a part in polyphenol biosynthesis. Recently, more attention has been paid to the PC of pear fruits, and several studies report these compounds could have a significant impact on the antioxidant capacity of pear fruit [3,4,5]. Phenolic acids and glycosylated hydroquinone (arbutin) are the two main groups of phenolic compounds in pear fruits [6,7]. However, the chemical composition of pear fruits and their products varies widely and is influenced by a series of factors such as cultivar, agroecological conditions, growing season, maturity stage, harvesting method, and storage conditions [8,9,10,11]. High-intensity agriculture currently relies on the cultivation of few genetically uniform cultivars, which yield fruits optimal and thus satisfy commercial needs. This trend has led to a global fruit landscape with standardized products, as well as a loss of traditional varieties and biodiversity. Traditional cultivars are particularly suited to zero-mile markets (from field to fork), contributing to improving product properties, protecting biodiversity, improving production sustainability, and safeguarding local traditional food [12]. As buying habits of environmentally conscious consumers have progressively changed, there is an enhanced demand for healthy food. It is generally accepted that local, old, traditional, autochthonous fruit cultivars have additional value in terms of the ability to adapt to different local ecological conditions. Moreover, recent studies have reported a significantly higher concentration of PC among traditional cultivars compared to commercial ones [6,13]. Traditional pear cultivars from Bosnia and Herzegovina possess several advantageous traits [14,15], including important morphological, pomological, eco-physiological, and nutritional characteristics of the fruit [1,16,17]. Local pear genotypes are highly appreciated by consumers due to their use in processing high-value fruit products (juices, nectars, dried fruit, purees, ‘pekmez’, and jams). These fruit cultivars are also important for the social and economic development of the rural area. In addition, they promote the diversification of agricultural production. Further knowledge of these old cultivars could therefore improve the conservation of this germplasm and processing in food industry and craft, thus making them more competitive. This study therefore aims to (i) determine individual sugars and organic acids in traditional pear cultivars, (ii) examine the polyphenol compounds in traditional pear cultivars, (iii) investigate the influence of cultivar and growing year on fruit quality parameters, and (iv) analyze the relationship among cultivars, growing season, part of fruit, and chemical parameters of pear. It is important to note that this is the first study to investigate organic acid, sugars, and polyphenol compounds of traditional pear cultivars from Bosnia and Herzegovina. Additionally, the study presented here examines traits vital both for fresh consumption as well as for fruit processing, thus potentially providing valuable recommendations for several stakeholders.

## 2. Materials and Methods

### 2.1. Plant Material

The research was conducted during two seasons. Meteorological data during the analyzed period are shown in Table 1. Pear fruit for the analysis was taken from an ex situ collection orchard located in Srebrenik (44°45′ N 18°28′ E; altitude 166 m). Based on a sensory evaluation of 29 pear cultivars by Alihodzic et al. [18], nine traditional pear cultivars were selected (‘Ahmetova’, ‘Budaljača’, ‘Dolokrahan’, ‘Hambarka’, ‘Kačmorka’, ‘Krakača’, ‘Ljeskovača’, ‘Sarajka’, and ‘Takiša’) as well as commonly grown ‘Président Drouard’ which served as a standard commercial cultivar, in this study. Pear cultivars were planted in 2002 on a *Pyrus communis* rootstock. The tree spacing was 4.0 × 2.0 m. The orchard was managed according to standard commercial practice for integrated fruit production (i.e., pruning, spraying, irrigation, etc.). The fruit was harvested at the technological maturity stage, which was determined using the starch iodine test from previously marked trees. Sugars and organic acids were analyzed in the whole fruit. Five replications were conducted for each cultivar (*n* = 5), each repetition including 15 pears sampled from five trees. All samples were immediately frozen in liquid nitrogen and kept at a temperature of −20 °C for further extraction and analysis.

### 2.2. Solvents and Reagents

Chemical standards of the carbohydrates (fructose, glucose, sucrose, and sorbitol) and organic acids (malic, citric, fumaric, and shikimic acids) were obtained from Fluka (Buchs, Switzerland) except malic acid, which was sourced from Merck (Darmstadt, Germany). The following standards were used for the quantification of individual polyphenolic compounds: chlorogenic acid (5-caffeoylquinic acid) and (−)-epicatechin from Sigma (St. Louis, MO, USA), (+)-catechin from Roth (Karlsruhe, Germany), quercetin 3-*O*-glucoside, quercetin 3-*O*-rutinoside, and arbutin from Fluka (Buchs, Switzerland). Methanol, acetonitrile of chromatographic grade quality, and butylhydroxytoluene (BHT) were purchased from Sigma–Aldrich (Steinheim, Germany). Deionized water was obtained using the Milli-Q system (Millipore, Billerica MA, USA).

### 2.3. Analysis of Sugars and Organic Acids

Individual sugars and organic acids were extracted from the whole pear fruit, and the same sample was used for the analysis of both sugars and organic acids. Ten grams of fresh fruits were used for each of the analyses. The tissue samples were increased in volume to 50 mL with bidistilled water, homogenized at room temperature with a T-25 Ultra-Turrax (IKA—Labortechnik, Staufen, Germany), and clarified by centrifugation at 10,000 rpm for 7 min at 5 °C (Thermo Scientific SL16 Centrifuge Series, San Jose, CA, USA). Before injection into the column, the samples were filtered through a 0.45 µm acetate cellulose filter (Macherey—Nagel, Düren, Germany) into vials. Samples of 20 µL of extract were used for the analysis of sugars and organic acids. Solute elution was monitored using the refractive index (RI).

The experimental procedure used to quantify the content of organic acids and sugars in pear extracts is the one adapted by Hudina et al. [19]. The Thermo Scientific Finnigan Surveyor HPLC system (Thermo Scientific, San Jose, CA) was used. Sugars were analyzed isocratically using a Hi-Plex Ca column (7.7 × 300 mm; 8 µm; Agilent Technologies, Santa Clara, CA, USA) with a temperature of 85 °C at a flow rate of 0.6 mL min^−1^, with bidistilled water used as the eluent.

Organic acids were analyzed using a Hi-Plex H column (7.7 × 300 mm; Agilent Technologies, Santa Clara, CA, USA) at 65 °C with a flow rate of 0.6 mL min^−1^. A UV detector was used with a wavelength set at 210 nm. For the mobile phase, 4 mM sulphuric acid was used.

The individual organic acids concentrations (malic, citric, shikimic, and fumaric acid) and sugars (sucrose, fructose, glucose, and sorbitol) were calculated using appropriate standards and expressed in g kg^−1^ of fresh fruit weight (FW). Total sugars (TS) and total organic acids (TA) were obtained by summing the concentrations of the individual components. The sugar/organic acid ratio (S/A) was calculated according to TS and TA. The glucose and fructose ratio (G/F) was calculated, too.

### 2.4. Analysis of Individual PC

Five independent extractions were carried out using 10 g (FW) of pulp or 5 g of peel, which were homogenized with a 10 mL extraction solution (methanol containing 3% formic acid and 1% *m*/*v* of BHT) in an ultrasonic ice bath (Elmasonic S 69 H; Elma Schmidbauer, Germany) for 1 h before centrifuging at 10,000 rpm for 7 min at 0 °C. The supernatant was filtered through a Chromafil AO-45/25 polyamide filter (Macherey–Nagel, Düren, Germany) into a vial. The analysis of phenolic compounds was carried out using a Thermo Scientific Finnigan Surveyor HPLC instrument (Thermo Scientific, San Jose, CA, USA). A Pursuit XRs 3 C-18 column (150 × 4.6 mm; Agilent Technologies, Santa Clara, CA, USA); particle size of five μm maintained at 25 °C was used for the separation. The spectra of phenolic compounds were also recorded between 210 and 400 nm [20]. The phenolic compounds were identified by their retention times and the use of external standards. The mobile phase consisted of aqueous 97% acetonitrile with 3% bidestilled water and 0.1% formic acid (A) and 97% bidestilled water with 3% acetonitrile and 0.1% formic acid (B) with the flow rate maintained at 0.6 mL min^−1^ and an injection amount of 20 μL. Phenolic compounds were determined using a photodiode array detector (PAD). These compounds were detected on the following wavelengths: 280 nm—chlorogenic acid, arbutin, (+) catehin, and (−) epicatehin, and at 350 nm—quercetin 3-*O*-glucoside and quercetin 3-*O*-rutinoside. Individual PC were calculated using the corresponding external standard and were expressed in mg kg^−1^ of fresh fruit weight. The sum of detected individual phenolics was calculated.

### 2.5. Statistical Analysis

Statistical data processing was performed using Statgraph 3.14 and SPSS 20 programs. The results were compared with a two-factor analysis of variance (ANOVA), and the established differences in mean values were tested by Tukey’s test at a significance level of 0.05. Principal component analysis (PCA) was used to identify the differentiation factor of pear fruit from cultivar and growing season based on analyzed chemical properties.

The visualization of overall parameters was obtained by a heatmap function. The heatmap was plotted by using the ClustVis program package (https://biit.cs.ut.ee/clustvis/online, accessed on 10 February 2022) and clustering both rows and columns with correlation distance and complete linkage. The sequential palette evidence the numerical differences of the data matrix: blue and red colors indicate lower and higher values, respectively.

## 3. Results and Discussion

### 3.1. The Content of Individual and Total Sugars

Sugars, together with organic acids, are the primary constituents of chemical fruit content, including pears. In light of this, the knowledge of sugar and acid profile as well as their relationship is very important in the food supply chain, from farmer to consumer. The content of these compounds is very important because sugar and organic acids, together with trace elements, determine the sensory and nutritional fruit quality [21]. Fruits with a higher content of sugar and organic acids, as well as optimal mineral content, are considered better quality and, as such, are more suitable for storage. Total and individual sugars are responsible for fruit juice sweetness. The individual sugar concentrations of fruit pulp therefore represent important information in terms of the authenticity of fruit juices. It is also an important component of the chemical composition tables [22]. The data on total and individual sugars in traditional and commercial pear cultivars in two growing years is shown in Table 2. A significant influence of cultivars, growing years, and their interaction was detected among the pear cultivars based on the content of analyzed individual and total sugars. The exception was the G/F ratio, among which significant influence of experimental factors and their interaction were not established. In all pear fruit samples, traditional and commercial, the concentration of fructose was the highest, followed by sorbitol and glucose, while the sucrose content was the lowest. These values are probably due to high invertase activity during the final ripening stage. The obtained results are in accordance with several previous studies on pear cultivars [23,24,25]. The G/F ratio ranged from 0.299 to 0.304, with slight variations. Similar results were reported by Dietrich et al. [26], who noted that the G/F ratio was in a range from 0.22 to 0.26 in analyzed cloudy pear juices produced from three cultivars. Wu and collaborators [27] established a sugar model to predict the distribution of carbon to sucrose, glucose, fructose, and sorbitol in peach fruit mesocarp, taking into account normal and high G/F ratios. According to this study, the extended model assumes a high G/F ratio to be caused by the preferential transformation of sorbitol into glucose, preferential utilization of fructose, or preferential conversion of fructose into glucose. In terms of this model, it could be predicted that the low G/F ratio in the analyzed pear cultivars is due to the preferential transformation of sorbitol into fructose, preferential utilization of glucose, or preferential conversion of glucose into fructose. For diabetics, the G/F ratio in fruit is very important as it helps keep the blood-sugar level constant [28]. The lowest content of sucrose was observed in the ‘Budaljača’ cultivar (4.0 g kg^−1^), followed by ‘Krakača’ (4.06 g kg^−1^) in the second growing season, and ‘Budaljača’, ‘Dolokrahan’ (4.42 g kg^−1^ and 4.54 g kg^−1^, respectively) both in the first growing season and these fruits could certainly be recommended in a low-sugar diet. In many fruits and fruit juices, sorbitol contributes significantly to the sugar-free extract. Its content is typical for a fruit species and shows a large range, from 0.15 g/L in fruit juice produced from black currant up to 65–100, 10–35, and 10–25 g/L in juice produced from aronia, sour cherry, and pear, respectively [26]. In view of that, sorbitol is a useful tool to indicate the authenticity of fruit juice.

It is important to note that the absorption of sorbitol into the bloodstream is usually incomplete and therefore does not increase blood glucose to a large extent as is the case with sucrose. Thus, sorbitol is popular as a sweetener in diabetics and people on a low-carbohydrate diet [29]. The average sorbitol content varied significantly among the years, ranging from 17.07 in the second season to 18.83 g kg^−1^ in the first one. The obtained results showed that the highest level of sorbitol was recorded in the first season, for all analyzed cultivars, except cv.’Dolokrahan’, ‘Takiša’, and ‘Ahmetova’. Among the analyzed pear cultivars, the average sorbitol content varied from 12.1 (‘Budaljača’) to 22.7 g kg^−1^ (‘Takiša’). The highest content was recorded by traditional cv. ‘Hambarka’ in the first growing season (25.4 g kg^−1^) and the lowest one by ‘Budaljača’ in the second one (11.5 g kg^−1^). Similar data for sorbitol content were reported by Hudina et al. [19], with specific differences due to the use of different pear cultivars and different climatic conditions, including water stress and temperature. Sorbitol is the main product of photosynthesis and represents 60–90% of all carbohydrates transported through the plant and is also a translocation substance of the genus *Pyrus* [30]. It is important to note that sorbitol has an important role in osmotic adjustment during water deficit. Namely, a higher content of sorbitol was detected in the first season, which had noticeably lower levels of precipitation during the maturing stage of the fruit (Table 1), foremost the months of August and September (0.8 and 35.5 mm, respectively), compared to the second season (26.5 and 60.4 mm, respectively). This is in agreement with results reported by Dietrich et al. [26], who investigated the content of sorbitol in pear juices produced from irrigated and non-irrigated trees. The authors found that pear juice produced from non-irrigated fruit had a higher sorbitol content (60.3 g/L) than juice from irrigated fruit (47.2 g/L).

The commercial pear cultivar ‘Président Drouard’ with three other traditional cultivars ‘Krakača’ ‘Dolokrahan’, and ‘Budaljača’ (74.3, 73.7, 68.1, and 55.6 g kg^−1^, respectively), had a lower content of total sugars than the other local ones. However, the highest content of total sugars was recorded in fruits from traditional cultivars ‘Hambarka’ and ‘Takiša’ (101.9 and 104.6 g kg^−1^, respectively).

### 3.2. The Content of Individual and Total Organic Acids

The content of total and individual organic acids, as well as the sugar/acid ratio of the investigated pear cultivars during two growing years, is presented in Table 3. A significant influence of cultivar, growing year, as well as their interaction, was established within the pear cultivars in terms of the content of analyzed individual and total organic acids. Fumaric and shikimic acid were the exceptions, among which significant differences were not established for the growing season or for the interaction of experimental factors in the case of fumaric acid. Malic and citric acids were the major organic acids, and malic acid was predominant in the examined pear cultivars. According to Kolniak-Ostek [11], the main organic acids were malic, citric, and shikimic acids, while malic acid was predominant in all investigated European pears. The total organic acid content of all cultivars ranged between 0.61 (‘Takiša’) and 3.89 (‘Krakača’) g kg^−1^ (Table 3).

In general, higher values of all examined organic acids were recorded in the first growing season. The concentration of total organic acids was higher in traditional pear cultivars, ranging from 1.24 to 3.89 g kg^−1^, compared to the commercial one (0.91 g kg^−1^), with the exception of traditional cultivars ‘Takiša’ and ‘Budaljača’, which had the lowest (0.61 g kg^−1^ and 0.73 g kg^−1^, respectively). The contents of malic and citric acid were higher than those of other acids, such as shikimic and fumaric, which is in accordance with the results published in a similar study [30]. The content of malic acid ranged from 0.35 g kg^−1^ (‘Dolokrahan’) in the second growing season to 2.8 g kg^−1^ (‘Krakača’) in the first, which is consistent with values for malic acid (from 1.42 to 2.67 g kg^−1^ FW) among commercial pear cultivars reported by Hudina et al. [19]. Citric acid was the second most abundant organic acid in pear cultivars, ranging from 0.14 g kg^−1^ FW (‘Dolokrahan’) in the second season to 2.6 g kg^−1^ (‘Krakača’) in the first. Fumaric acid and shikimic acid occurred in minor amounts when compared with other analyzed acids. The highest content of fumaric acid was detected in the traditional cultivar ‘Krakača’ (0.01 g kg^−1^) harvested in the first season, while the lowest content was found in fruits of the commercial cultivar ‘Président Drouard’ (0.001 g kg^−1^) harvested in the second growing season.

The sugar/acid (S/A) ratio is commonly used to determine the sensory quality, particularly the flavor and taste of fruit and fruit juices [31]. The S/A ratio of all cultivars ranged between 20.6 (‘Krakača’) and 172.7 (‘Takiša’). According to Lee et al. [32], apple cultivars with a S/A ratio of 20 and lower are sharp and appropriate for processing and cider production, while cultivars with a higher S/A ratio than 20 are sweet and good for direct consumption. The same pattern can be applied to pear cultivars and, as can be seen from Table 3, all analyzed pear cultivars had S/A ratios higher than 20, being classified as sweet and good for direct consumption and processing into different fruit products characterized by a higher dry matter, such as ’’pekmez’’ jam, marmalade, etc. On the other hand, cv. ‘Krakača’ had the S/A ratios of about 20, making it suitable for juice processing.

### 3.3. Phenolics Composition

The content range of total polyphenols determined in different parts of pear fruit, among local and commercial cultivars, during two growing seasons is presented in Table 4. Some variations in polyphenols were noted among the ten pear cultivars and growing seasons, with few exceptions (Table 4). A total of six phenolic compounds were identified and quantified in pear peel and four compounds in pear pulp. The occurrence and distribution of the main polyphenol groups differed between peel and pulp, as reported previously by Li et al. [10], Kolniak-Ostek [11], and Azzini et al. [13]. Six phenolic compounds identified in pear peel belong to four major phenolic groups. They are hydroxycinnamic acid (chlorogenic acid), glycosylated hydroquinone (arbutin), flavan-3-ols (epicatechin and catechin), and flavonols (quercetin 3-*O*-glucoside and quercetin 3-*O*-rutinoside). The obtained results indicated that glycosylated hydroquinone and hydroxycinnamic acid are the main phenolic constituents in the analyzed pear cultivars, followed by flavan-3-ols and flavonols, which is consistent with results reported by Cui et al. [6] and Kiran et al. [7].

Cultivars ‘Kačmorka’ and ‘Sarajka’ were the exceptions, among which the phenol groups have the following order: glycosylated hydroquinone > flavan-3-ols > flavonols > hydroxycinnamic acid and glycosylated hydroquinone > flavonols > hydroxycinnamic acid > flavan-3-ols, respectively. The same phenolic subgroups were identified in the pulp, with the exception of flavonols. The content of all analyzed polyphenol compounds was higher in pear peel (574.93 mg kg^−1^) than in pulp (36.86 mg kg^−1^), which is in accordance with earlier reported results [10,33]. Traditional pear cultivars had higher total phenols in both peel and pulp than the commercial ‘Président Drouard’, with the exception of cultivars ‘Takiša’ and ‘Ahmetova’ (Table 4). Traditional pear cultivars such as ‘Dalokrahan’, ‘Krakača’, and ‘Budaljača’ possessed more than twice the content of total phenols (840.3, 1079.8, and 1147.8 mg kg^−1^, respectively) compared to the commercial cultivar (401.46 mg kg^−1^). The results are in agreement with those reported by Öztürk et al. [34], who found an increase in the content of phenols in autochthonous or local pear cultivars compared to commercial ones. A higher level of phenolic compounds in traditional cultivars could be a mechanism of plant response to biotic and abiotic stressors [16]. It is important to note that a higher content of analyzed polyphenol compounds was recorded in the first compared to the second season, with only a few exceptions. This was expected, as chlorogenic acid was one of the main phenolic constituents in the analyzed pear cultivars, and, according to the data shown above in Table 3, the content of total acids was higher in the first than in the second season. Additionally, the overall higher content of PCs in the first year could at least in part be due to higher levels of insolation, as well as higher, mean temperature detected during that growing season (Table 1). Chlorogenic acid, as reported earlier by Kolniak-Ostek [11], is important as a taste precursor in fruit and their products. Additionally, it is a strong antioxidant, which can eliminate superoxide radicals [35]. A great variation in terms of chlorogenic acid content was observed among cultivars, with the highest content recorded for ‘Krakača’, while ‘Kačmorka’ and ‘Sarajka’ had the lowest. The content of chlorogenic acid in the first growing season was higher than in the second, ranging from 11.94–359.96 mg kg^−1^ (peel) and 0.46–14.94 mg kg^−1^ (pulp) to 2.27–287.34 mg kg^−1^ (peel) and 0.10–9.69 mg kg^−1^ (pulp), respectively. The results confirm earlier reports about chlorogenic acid both in different parts of pear fruit and in growing years [25].

Arbutin (hydroquinone-β-D-glucopyranoside) is a naturally occurring glycoside of hydroquinone, and it is the primary phenolic compound in the different parts of the pear plant and fruit. It has attracted attention for its antibacterial, anti-inflammatory, and antitussive effects and is commonly used in urinary therapeutics and as a human skin-whitening agent in cosmetic products [36]. The highest amount of determined individual phenolic compounds in the examined pear cultivars during the two growing years was arbutin, which ranged from 75.07–647.40 mg kg^−1^ (peel, the first season) and 60.63–472.48 mg kg^−1^ (peel, the second one) to 4.34–59.83 mg kg^−1^ (pulp, the first season) and 3.0–66.05 mg kg^−1^ (pulp, the second). These observations were in disagreement with the results for the arbutin content in a previous study on European pear fruits, which reported a variation from 4.0 to 22.5 mg/100 g FW [37]. The arbutin content in seven Korean pear cultivars ranged from 6.3 ± 1.9 to 36.2 ± 5.2 mg/100 g FW [38]. In addition, in Asian pears, it ranged from 5 to 45 mg/100 g FW [6]. These observations indicate that the wide range of arbutin contents may be dependent on species and cultivars. Great variations of the arbutin content among peel and pulp were presumably due to different weather conditions during pear-growing years (Table 1), which had an influence on the biosynthesis of simple phenol compounds. The highest content of arbutin was recorded in traditional pear cultivars ‘Krakača’,‘Budaljača’, and ‘Sarajka’ in the first season, and Budaljača’ and ‘Dolokrahan’ in the second one, in both fruit peel and pulp. Flavan-3-ols, catechin, and epicatechin are generally located in the peel of pome fruit, which was confirmed in this study. According to Engler and Engler [39], these compounds were found to exhibit antioxidative, anticancer, and antibacterial properties. Generally, the catechin content was predominantly higher in peel in all analyzed pear cultivars, and it was up to seven-fold higher in the first season (5.63–47.71 mg kg^−1^) compared to the second season (0.45–22.19 mg kg^−1^). In the pear pulp of the analyzed cultivars, the average content of catechin ranged from 0.10 to 1.77 mg kg^−1^ (the first season) and 0.10–1.82 mg kg^−1^ (the second one). Catechin content was significantly the highest in traditional pear cultivars ‘Budaljača’ and ‘Krakača’ (in the peel) and in ‘Budaljača’ (in pulp) during the analyzed growing years. The level of epicatechin in the peel of all analyzed pear cultivars was higher than in pulp, which is consistent with the results obtained by Li et al. [10]. The highest content of epicatechin in peel was recorded for ‘Krakača’ (350.28 mg kg^−1^) in the first season and ‘Budaljača’ (112.91 mg kg^−1^) in the second one. Traditional cultivars ‘Kačmorka’ and ‘Sarajka’ had the lowest content of epicatechin in peel among the analyzed pear cultivars during the first season and ‘Président Drouard’ in the second growing year. Flavonols, quercetin 3-*O*-glucoside, and quercetin 3-*O*-rutinoside were recorded in the lowest amount among the investigated pear cultivars, with some exceptions. These compounds were identified only in peel for the analyzed pear cultivars. This corresponds to data obtained by Renard et al. [40], showing that flavonols were absent or at trace level only in the flesh of analyzed pome fruits but in much higher contents in the peel, and their contents decreased in fruits for cider production but increased in table fruits. Table 4 shows that, of the two flavonols, quercetin 3-*O*-glucoside was the most abundant in both traditional and commercial pear cultivars. The highest amount of quercetin 3-*O*-glucoside was detected in pear samples of the cultivar ‘Sarajka’ (125.39 mg kg^−1^) in the first season, and ‘Budaljača’ and ‘Président Drouard’ (25.32 and 31.95 mg kg^−1^, respectively) in the second one. Quercetin is an important phenol with antioxidative properties, although it is much more easily taken up in the human body in the form of glucosides, which are afterward transformed into quercetin. The amount of quarcetin 3-O-glycosides could therefore be important for the nutritional value of pome fruit [32].

### 3.4. PCA and Heatmap Analysis

**Figure 1:**
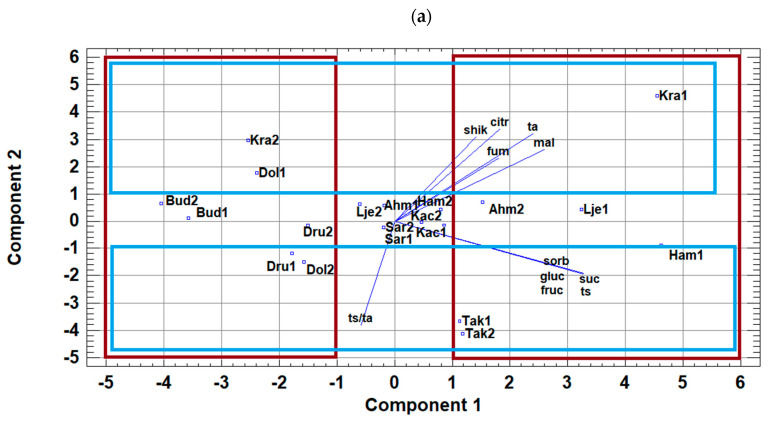

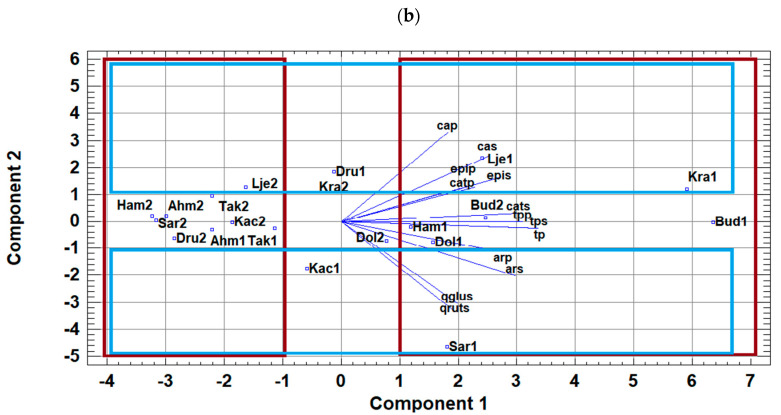
Principal component analysis (PCA) plots presenting graphical representation of the position of the analyzed pear cultivars from different growing seasons in relation to (**a**) analyzed sugars (gluc—glucose; fruc—fructose; suc—sucrose; ts—total sugars) and organic acids (ta—total acids; citr—citric; shik—shikimic; fum—fumaric; mal—malic acid; ts/ta—sugar/acid ratio) and (**b**) polyphenol compounds (cap—chlorogenic acid into pulp; cas—chlorogenic acid into skin; epip—epicatechin into pulp; epis—epicatechin into skin; catp—catechin into pulp; cats—catechin into skin; tpp—total polyphenols into pulp; tps—total polyphenols into skin; arp—arbutine into plulp; ars—arbutine into skin; qglus—quercetin 3-*O*-glucoside into skin and qruts—quercetin 3-*O*-rutinoside into skin) (pear cultivars: Kra—‘Krakača’, Dol—‘Dolokrahan’, Bud—‘Budaljača’, Dru—‘Président Drouard’, Kac—‘Kačmorka’, Sar—‘Sarajka’, Ahm—‘Ahmetova’, Lje—‘Ljeskovača’, Tak—‘Takiša’, and Ham—‘Hambarka’; growing season: 1, 2).

According to the results of the principal component analysis (PCA), the variability of sugar and acid content is largely explained by two principal components (87.09% of the total variability), with the first component accounting for 52.05% of the overall variability. Figure 1a shows the PCA score plot on sugars and organic acids of pear, whereby the distinction between cultivars (traditional and commercial ones) and growing season can be seen.

It is clear that traditional cultivars ‘Takiša’ in both years, and ‘Krakača’, ‘Ljeskovača’, and ‘Hambarka’ in the first season, as well as ‘Ahmetova’ (second season), are located in the positive part of the PC1 component and were dominantly determined with all analyzed individual and total sugars and organic acids. Cultivar ‘Krakača’ was predominantly defined by the greatest content of organic acids while cv. ‘Takiša’ was determined by the highest content of sugars as well as the S/A ratio.

On the other hand, ‘Krakača’ (second season) and ‘Dolokrahan’, ‘Budaljača’, and ‘Président Drouard’ in both years are positioned in the negative part of the PC1 component and were determined with a lower content of the mentioned compounds. In terms of the growing season, all the analyzed pear cultivars were distinguished, with the exception of cvs. ‘Kačmorka’, ‘Sarajka’, ‘Takiša’, and ‘Budaljača’.

Figure 1b shows the score plot of PCA on the polyphenol compounds of pear, in which the distinction between parts of fruit (peel and pulp), cultivars (traditional and commercial ones), and growing season can be seen. The variability of polyphenolics is explained by three principal components: 85.13% of total variability and two components accounting for 77.2% of the variability.

Pear cultivars ‘Krakača’ and ‘Ljeskovača’ in both years and ‘Président Drouard’ in the first season were located in the positive part of PC1 and PC2 components. It means that these cultivars were determined by a higher content of chlorogenic acid and epicatechin in both peel and pulp and catechin content in the pulp. Cultivars ‘Sarajka’ and ‘Kačmorka’ in the first season were determined by arbutin (in peel and pulp) as well as flavonol’s content.

**Figure 2:**
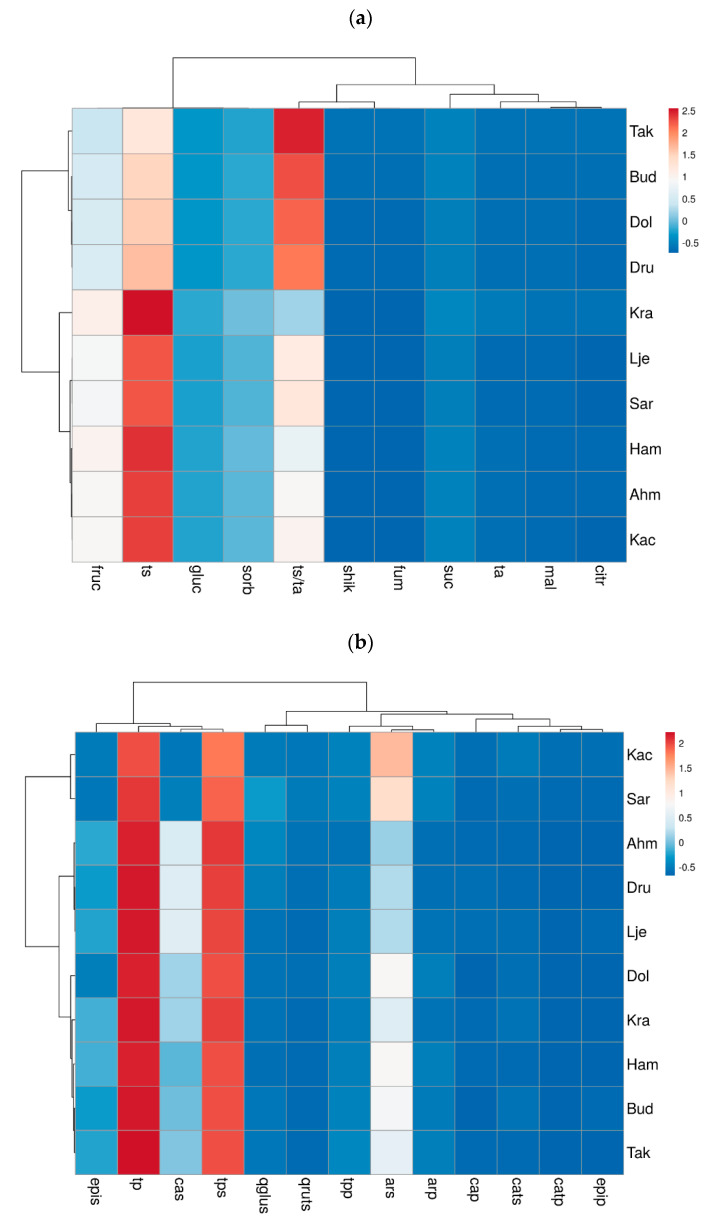
Heatmap of (**a**) sugars and organic acids and (**b**) polyphenol compounds detected in 10 different pear cultivars based on the relative abundance of different compounds.

A heatmap was produced for the analyzed parameters in order to provide a detailed overview of the average individual and total sugars, organic acids, S/A ratio, as well total and individual polyphenols in the skin and pulp of different pear varieties in two years (Figure 2). The agglomerative hierarchical clustering (AHC) of pear varieties grouped samples by their dissimilarity into two main clusters: cluster I with only ‘Takiša’, ‘Budaljača’, ‘Dolokrahan’, and ‘Président Drouard’; and cluster II containing ‘Krakača’, ‘Ljeskovača’, ‘Sarajka’, ‘Hambarka’, ‘Ahmetova’, and ‘Kačmorka’ (Figure 2a).

The biggest dissimilarities among the 10 cultivars were found in the sugar/acid ratio, total sugar, and fructose. The AHC of pear varieties grouped samples into two main clusters. Cluster I included ‘Ahmetova’, ‘Président Drouard’, ‘Hambarka’, ‘Kačmorka’, ‘Sarajka’, ‘Ljeskovača’, and ‘Takiša’, and the second cluster contained only the varieties ‘Budaljača’, ‘Krakača’ and ‘Dolokrahan’ (Figure 2b). The clustering of the analyzed polyphenols shows the biggest dissimilarities among pear varieties in relation to arbutin, chlorogenic acid, epicatechin, total phenols in the skin, and total phenols in whole fruit.

## 4. Conclusions

The obtained results of this research showed a significant influence of climatic conditions on the quality of pear fruits. Significant differences were found between sugars, organic acids, and polyphenol compounds depending on year and cultivar. In general, it can be concluded that traditional cvs. ‘Takiša’, ‘Hambarka’, ‘Ahmetova’, ‘Krakača’, and ‘Ljeskovača’ differentiated among the analyzed cultivars in terms of individual and total sugars, organic acids, and S/A. The dominant phenolic compounds in pear fruit samples were arbutin and chlorogenic acid. The extremely high polyphenol content in the cvs. ‘Budaljača’, ‘Dolokrahan’, and ‘Krakača’, in both growing seasons, highlights their value for use as both a source of fresh fruit and raw material for processing. These traditional pear cultivars can also be used for nutrition, enriching different fruit products made from commercial pear cultivars, as well improving their sensory attributes. The obtained results can contribute to the development of strategies and the creation of new products in the nutraceutical, pharmaceutical, and food industries. However, further detailed assessment of traditional pear varieties is needed in relation to the most important properties to promote the valorization of niche markets, as well as their dissemination and conservation.

## Figures and Tables

**Table 1:** Average values of weather parameters during the pear-growing season (PGS).

Weather Season Parameters		Annual Average	Pear-Growing Season (Calendar Months)							Average PGS
			IV	V	VI	VII	VIII	IX	X	
Minimum T (°C)	1	8.7	8.4	12.6	17.6	19.8	19.7	14.5	8.6	14.5
	2	8.6	9.1	12.6	16.2	19	18.7	12.6	9.9	14.0
Maximum T (°C)	1	18.2	18.8	21.9	28.3	31.2	32.1	26	18.7	25.3
	2	17.1	18.5	21.8	24.5	28.8	29.2	22	20.1	23.6
MeanT (°C)	1	13.3	13.3	16.8	22.8	25.2	25.8	20.1	13.3	19.6
	2	12.8	14.1	16.6	20.4	23.6	23.7	16.9	15.1	18.6
Insolation (hours)	1	2395.4	189	255.2	333.4	335.8	356	221	159.5	264.3
	2	2178	241	215.6	252.5	375.3	299.7	188.6	189.3	251.7
Cloudiness (C 0–8)	1	4	5	4	3	3	2	4	5	3.7
	2	5	4	5	4	3	3	5	4	4.0
Precipitation (mm)	1	721.7	111.4	121.3	89.7	44.5	0.8	35.5	74	68.2
	2	846.6	54.5	175.1	86.6	35.9	26.5	60.4	77.3	73.8
Rainfall days *	1	96	14	14	7	7	1	5	12	60
	2	124	9	17	12	6	7	12	6	69
Snowy days *	1	27	1	0	0	0	0	0	0	1.0
	2	27	1	0	0	0	0	0	0	1.0
Hazy days *	1	0	0	0	0	0	0	0	0	0.0
	2	1	0	1	0	0	0	0	0	1.0
Foggy days *	1	27	0	0	0	0	0	0	8	8.0
	2	48	2	0	0	0	1	3	3	11
Windy days *	1	37	6	7	3	1	1	0	2	20
	2	11	0	2	0	0	0	1	0	3.0

* Average of pear-growing season during analyzed period; data from Federal Hydrometeorological Service, Sarajevo (B&H).

**Table 2:** Average content of individual sugars, total sugars (TS), and glucose/fructose ratio (G/F) ± SD in analyzed pear cultivars (g kg^−1^) FW*.

Sugar/ Growing Season		‘Dolokrahan’	‘Takiša’	‘Ahmetova’	‘Ljeskovača’	‘Hambarka’	‘Budaljača’	‘Kačmorka’	‘Krakača’	‘Sarajka’	‘Président Drouard’
Sucrose	1	4.54 ± 0.17 ef	7.9 ± 0.16 ab	6.04 ± 0.3 de	7.7 ± 0.004 abc	8.9 ± 0.02 a	4.42 ± 0.3 f	6.82 ± 0.2 bcde	7.16 ± 0.2 bcd	6.6 ± 0.2 cde	5.71 ± 1.2 e
	2	5.91 ± 0.06 bc	8.0 ± 0.45 a	6.85 ± 0.18 ab	5.77 ± 0.5 bc	6.61 ± 0.53 ab	4.0 ± 0.51 c	6.58 ± 1.1 ab	4.06 ± 0.3 c	6.3 ± 1.4 ab	5.59 ± 0.7 bc
	*Xs*	*5.22 ± 0.75 de*	*7.96 ± 0.31 a*	*6.45 ± 0.51 cd*	6.75 ± 1.12 abc*	7.75 ± 1.29 ab*	*4.22 ± 0.42 e*	*6.70 ± 0.75 bc*	5.61 ± 1.71 cd*	*6.46 ± 0.90 cd*	*5.65 ± 0.88 cd*
Glucose	1	9.72 ± 0.36 de	17.1 ± 0.3 ab	12.9 ± 0.7 cd	16.8 ± 0.4 abc	19.1 ± 0.04 a	9.5 ± 0.54 e	14.6 ± 0.4 bcd	15.3 ± 0.4 bc	14.2 ± 0.4 cd	12.2 ± 2.6 d
	2	12.6 ± 0.13 bc	17.2 ± 0.9 a	14.7 ± 0.39 ab	12.5 ± 1.1 bc	14.2 ± 1.15 ab	8.64 ± 1.1 c	14.1 ± 2.5 ab	8.70 ± 0.6 c	13.5 ± 2.9 ab	12.0 ± 1.5 bc
	Xs	*11.2 ± 0.13 de*	*17.1 ± 0.97 a*	*13.8 ± 0.39 c*	14.7 ± 1.06 abc*	*16.6* ± 1.14 ab*	*9.06 ± 1.09 e*	*14.4 ± 2.45 bc*	12.0 ± 0.57 cd*	*13.8 ± 2.97 bc*	*12.1 ±1.50 cd*
Fructose	1	32.0 ± 1.4 ef	56.4 ± 1.1 ab	43.2 ± 2.4 de	55.5 ± 0.5 abc	63.6 ± 0.1 a	31.6 ± 1.8 f	48.7 ± 1.4 bcd	51.2 ± 1.3 bcd	47.3 ± 1.4 cde	40.8 ± 8.6 de
	2	41.6 ± 1.2 bc	57.3 ± 3.2 a	48.9 ± 1.3 ab	41.4 ± 3.5 bc	47.2 ± 3.8 ab	28.8 ± 3.7 c	47.0 ± 8.2 ab	29.0 ± 1.9 c	45.1 ± 9.9 b	39.9 ± 5.0 bc
	*Xs*	*36.8 ± 1.23 cd*	*56.8 ± 3.23 a*	*46.0 ± 1.31 b*	48.5 ± 3.50 b*	55.4 ± 3.82 ab*	*30.2 ± 3.67 d*	*47.9 ± 8.18 b*	40.1 ± 1.90 bc*	*46.2 ± 9.91 b*	*40.4 ±4.98 bc*
Sorbitol	1	12.9 ± 0.5 g	22.6 ± 0.5 ab	17.3 ± 1.0 de	22.4 ± 0.5 abc	25.4 ± 0.05 a	12.6 ± 0.7 g	19.5 ± 0.6 bcde	20.5 ± 0.5 bcd	18.9 ± 0.6 cde	16.3 ± 3.5 ef
	2	16.8 ± 0.3 bc	22.9 ± 1.3 a	19.6 ± 0.5 ab	16.7 ± 1.4 bc	18.9 ± 1.5 ab	11.5 ± 1.5 c	18.8 ± 3.3 ab	11.6 ± 0.8 c	18.0 ± 4.0 ab	16.0 ± 2.0 bc
	*Xs*	*14.9 ± 0.26 cd*	*22.7 ± 1.29 a*	*18.4 ± 0.52 b*	19.6 ± 1.41 ab*	22.1 ± 1.53 ab*	*12.1 ± 1.46 d*	*19.2 ± 3.27 b*	16.0 ± 0.76 bc*	*18.5 ± 3.96 b*	*16.1 ± 1.99 bc*
Total sugar	1	59.2 ± 2.33 fg	103.8 ± 2.7 ab	79.4 ± 4.42 de	102.5 ± 1.49 abc	116.9 ± 0.24 a	58.1 ± 3.36 g	89.7 ± 2.66 bcde	94.1 ± 2.47 bcd	86.9 ± 2.63 cde	75.0 ± 15.87 ef
	2	76.9 ± 1.64 bc	105.4 ± 5.94 a	90.0 ± 2.4 ab	76.4 ± 6.44 bc	86.8 ± 7.02 ab	53.0 ± 6.74 c	86.5 ± 3.49 ab	53.4 ± 3.49 c	82.9 ± 18.22 ab	73.5 ± 9.17 bc
	*Xs*	68.1 ± 9.91 de*	*104.6 ± 4.07 a*	84.7 ± 6.64 cd*	89.6 ± 14.87 b*	101.9 ±17.08 ab*	*55.6 ± 5.52 e*	*88.2 ± 9.82 bc*	73.7 ± 22.49 de*	*85.0 ± 11.86 c*	*74.3 ± 11.62 d*
G/F ratio	*Xs*	*0.304 ± 0.0062*	*0.301 ± 0.00*	*0.300 ± 0.00*	*0.303 ± 0.0039*	*0.300 ± 0.00*	*0.300 ± 0.00*	*0.301 ± 0.00*	*0.299 ± 0.00*	*0.299 ± 0.00*	*0.300 ± 0.00*

Average values ± standard deviation (SD) in rows marked with different letters (a–g) represent statistically significant differences between pear cultivars; an asterisk indicates statistically significant differences in the same pear cultivar through different years; Tukey’s test, *p* < 0.05.

**Table 3:** Average content of individual organic acids, total acids (TA), and sugars/acids ratio (S/A) in analyzed pear cultivars (g kg^−1^) FW*.

Organic Acids Season		‘Dolokrahan’	‘Takiša’	‘Ahmetova’	‘Ljeskovača’	‘Hambarka’	‘Budaljača’	‘Kačmorka’	‘Krakača’	‘Sarajka’	‘Président Drouard’
Malic acid	1	1.52 ± 0.1 c	0.50 ± 0.1 ef	0.77 ± 0.2 de	1.92 ± 0.2 b	2.08 ± 0.04 b	0.31 ± 0.1 f	1.28 ± 0.1 c	2.8 ± 0.2 a	1.14 ± 0.1 cd	0.40 ± 0.08 ef
	2	0.35 ± 0.1 d	0.40 ± 0.1 d	1.38 ± 0.2 a	0.57 ± 0.1 cd	0.83 ± 0.1 bc	0.4 ± 0.002 d	1.33 ± 0.05 a	1.1 ± 0.1 ab	1.10 ± 0.01 ab	0.87 ± 0.1 b
	*Xs*	0.93 ± 0.65 e*	*0.44 ± 0.11 fg*	1.07 ± 0.37 de*	1.25 ± 0.74 bcd*	1.46 ± 0.69 b*	*0.35 ± 0.1 g*	*1.35 ± 0.9 bc*	1.96 ± 0.84 a*	*1.12 ± 0.02 cde*	0.64 ± 0.2 f*
Citric acid	1	0.35 ± 0.02 cd	0.20 ± 0.01 d	0.67 ± 0.2 b	0.80 ± 0.07 b	0.55 ± 0.1 bc	0.38 ± 0.01 cd	0.4 ± 0.001 cd	2.6 ± 0.2 a	0.70 ± 0.03 cd	0.20 ± 0.07 d
	2	0.14 ± 0.04 e	0.16 ± 0.4 de	0.97 ± 0.1 a	0.33 ± 0.05 c	0.81 ± 0.06 b	0.3 ± 0.004 cd	0.35 ± 0.05 c	1.0 ±0.03 a	0.40 ± 0.01 c	0.24 ± 0.1 cde
	*Xs*	0.25 ± 0.12 de*	*0.15 ±0.02 e*	0.82 ± 0.21 b*	0.57 ± 0.23 c*	0.68 ± 0.16 bc*	*0.34 ± 0.38 d*	*0.36 ± 0.04 d*	1.82 ± 0.8 a*	*0.36 ±0.03 d*	*0.23 ± 0.05 de*
Shikimic acid	1	0.06± 0.004 b	0.02 ± 0.01 d	0.06 ± 0.01 bc	0.07 ± 0.01 b	0.05 ± 0.007 bcd	0.03 ± 0.005 cd	0.08 ± 0.008 b	0.15 ± 0.02 a	0.02 ± 0.004 d	0.04 ± 0.008 cd
	2	0.06 ± 0.005 abc	0.02 ± 0.001 c	0.05 ± 0.004 bc	0.11± 0.02 a	0.07 ± 0.01 abc	0.04± 0.01 bc	0.08 ±0.005 ab	0.05 ± 0.007 bc	0.059 ± 0.04 abc	0.049 ± 0.009 bc
	*Xs*	*0.06 ± 0.004 bcd*	*0.02 ± 0.006 e*	*0.06 ± 0.01 cd*	*0.09 ± 0.02 ab*	*0.06 ± 0.02 cd*	*0.04 ± 0.01 de*	*0.08 ± 0.005 abc*	*0.100 ± 0.05 a*	*0.04 ± 0.03 de*	*0.04 ±0.01 de*
Fumaric acid	1	0.003 ± 0.001 c	0.0016 ± 0.0008 c	0.008 ± 0.0006 ab	0.009 ± 0.002 a	0.008 ± 0.001 ab	0.004 ± 0.0008 bc	0.003 ± 0.002 c	0.01 ± 0.002 a	0.0039 ± 0.0008 bc	0.003 ± 0.0007 c
	2	0.003 ± 0.0001 cde	0.002 ± 0.0006 de	0.007 ± 0.001 ab	0.009 ± 0.002 a	0.006 ± 0.001 bc	0.005 ± 0.0005 bcd	0.004 ± 0.001 cd	0.007 ± 0.001 b	0.004 ± 0.0001 cd	0.001 ± 0.0001 e
	*Xs*	*0.003 ± 0.001 d*	*0.002 ± 0.0006 a*	*0.007 ± 0.0009 b*	*0.009 ± 0.001 a*	*0.007 ± 0.001 b*	*0.004 ± 0.0006 c*	*0.004 ± 0.001 cd*	*0.008 ± 0.002 ab*	*0.004 ± 0.0004 cd*	*0.002 ± 0.001 d*
Total acid	1	1.94 ± 0.16 c	0.66 ± 0.09 d	1.51 ± 0.34 c	2.81 ± 0.15 b	2.69 ± 0.07 b	0.72 ± 0.15 d	1.83 ± 0.13 c	5.61 ± 0.22 a	1.53 ± 0.11 c	0.67 ± 0.05 d
	2	0.55 ± 0.09 e	0.56 ± 0.08 e	2.4 ± 0.13 a	1.01 ± 0.15 cd	1.72 ± 0.08 b	0.75 ± 0.01 de	1.76 ± 0.09 b	2.18 ± 0.12 a*	1.52 ± 0.01 b	1.15 ± 0.18 c
	*Xs*	1.24 ± 0.76 e*	*0.61 ± 0.09 g*	1.96 ± 0.54 bc*	*1.92 ± 0.98 c*	*2.20 ± 0.55 b*	*0.73 ± 0.09 gf*	*1.79 ± 0.11 dc*	*3.89 ± 1.78 a*	*1.52 ± 0.07 ed*	*0.91 ± 0.29 f*
Sugar/acid ratio	1	15.05 ± 1.81 de	157.3 ± 22.74 a	52.58 ± 8.88 cd	36.48 ± 1.56 de	43.46 ± 1.10 de	80.7 ± 12.23 bc	49.02 ± 5.07 d	16.8 ± 1.11 e	56.8 ± 5.51 cd	111.9 ± 17.08 b
	2	139.8 ± 28.96 b	188.2 ± 18.64 a	37.5 ± 2.08 de	75.64 ± 15.82 c	50.47 ± 6.93 de	70.67 ± 8.13 c	49.2 ± 10.16 de	24.5 ± 1.19 e	54.5 ± 11.83 de	63.9 ± 15.06 de
	*Xs*	77.43 ± 63.85 b*	*172.7 ± 25.61 a*	*45.04 ± 10.7 cd*	56.06 ± 24.28 cd*	*46.96 ± 6.02 cd*	*75.7 ± 11.02 a*	*49.08 ± 7.18 cd*	*20.6 ± 4.32 d*	*55.7 ± 8.38 cd*	*87.9 ± 29.07 c*

Average values ± standard deviation (SD) in rows marked with different letters (a–g) represent statistically significant differences between cultivars; an asterisk indicates statistically significant differences in the same pear cultivar through different years; Tukey’s test, *p* < 0.05.

**Table 4:** Content of phenolic compounds and total phenols (mg kg^−1^) in different parts of pear fruit cultivars during two growing years.

	Phenolics	Season	Cultivars									
			‘Dolokrahan’	‘Takiša’	‘Ahmetova’	‘Ljeskovača’	‘Hambarka’	‘Budaljača’	‘Kačmorka’	‘Krakača’	‘Sarajka’	‘Président Drouard’
PEEL	Chlorogenic acid	1	241.7 ± 14.32 b	69.98 ± 4.61 de	114.85 ± 19.45 d	338.29 ± 10.02 a	175.51 ± 26.03 c	359.96 ± 29.60 a	11.94 ± 0.37 f	335.08 ± 6.47 a	26.42 ± 3.53 ef	255.70 ± 35.79 b
		2	243.25 ± 9.39a	91.09 ± 7.09d	95.88 ± 7.01cd	149.4 ± 25.56bc	33.74 ± 5.11ef	167.79 ± 44.13b	2.27 ± 0.47f	287.34 ± 29.55a	24.03 ± 1.33ef	68.87 ± 5.74de
		*Xs*	242.5 ± 104.9b	80.53 ± 12.7d*	105.37 ± 16.7d	243.8 ± 104.9b*	104.63 ± 79.4d*	263.9 ± 110.5b*	7.10 ± 5.3e*	311.21 ± 32.4a	25.23 ± 2.7e	162.3 ± 104.9c*
	Catechin	1	30.93 ± 2.59 b	7.67 ± 1.87 cd	5.63 ± 0.45 d	18.39 ± 1.29 bcd	7.20 ± 1.64 cd	47.62 ± 8.24 a	19.96 ± 0.53 bc	47.71 ± 6.63 a	9.57 ± 1.05 cd	7.88 ± 1.43 cd
		2	6.94 ± 1.20 bcd	2.68 ± 0.24 cde	0.45 ± 0.01 e	8.44 ± 2.51 bc	1.76 ± 0.34 de	22.19 ± 4.42 a	10.68 ± 2.02 b	10.49 ± 2.21 b	0.50 ± 0.01 e	2.73 ± 0.67 cde
		*Xs*	18.9 ± 13.26 b*	5.17 ± 3.28 c	3.04 ± 2.58 c*	13.41 ± 5.07 b*	4.48 ± 3.17 c*	34.90 ± 15.88 a*	15.32 ± 5.25 b*	29.1 ± 20.19 a*	5.03 ± 4.13 c*	5.31 ± 3.32 c*
	Epicatechin	1	93.07 ± 8.31 de	84.34 ± 10.59 de	66.01 ± 12.03 ef	139.2 ± 11.2 bcd	170.29 ± 24.60 bc	184.80 ± 22.27 b	12.56 ± 0.38 f	350.28 ± 34.41 a	20.17 ± 2.55 f	108.3 ± 17.6 cde
		2	14.42 ± 0.42 cd	19.46 ± 3.21 cd	21.37 ± 2.27 cd	37.85 ± 6.28 bc	20.11 ± 3.57 cd	112.91 ± 20.29 a	15.37 ± 3.92 cd	57.47 ± 9.72 b	14.11 ± 3.28 cd	1.84 ± 0.45 d
		*Xs*	53.7 ± 43.39 e*	51.90 ± 36.21 e*	43.69 ± 27.28 ef*	88.53 ± 56.4 cd*	95.2 ± 83.79 c*	148.86 ± 52.66 b	13.96 ± 2.93 f	203.9 ± 162.2 a*	17.14 ± 4.57 f	55.07 ± 49.37 de*
	Q-glucoside	1	42.15 ± 1.31 cd	29.02 ± 4.02 def	37.18 ± 3.7 de	28.43 ± 4.27 def	17.01 ± 2.92 f	64.31 ± 10.47 b	26.64 ± 1.34 def	56.23 ± 4.69 bc	125.39 ± 14.56 a	18.92 ± 2.13 ef
		2	11.33 ± 1.17 b	1.85 ± 0.43 c	6.45 ± 1.85 bc	7.22 ± 1.13 bc	0.46 ± 0.01 c	25.32 ± 4.88 a	0.41 ± 0.02 c	6.49 ± 1.24 bc	5.09 ± 0.66 bc	31.95 ± 6.35 a
		*Xs*	26.7 ± 16.9 cd*	15.44 ± 13.56 ef*	21.8 ± 17.08 cde*	17.8 ± 11.9 def*	8.74 ± 9.25 f*	44.81 ± 22.77 b*	13.5 ± 11.39 ef*	31.36 ± 27.42 c*	65.24 ± 46.53 a*	25.44 ± 8.30 cd*
	Q-rutinoside	1	16.35 ± 1.18 bc	11.63 ± 1.66 c	11.11 ± 1.15 c	13.43 ± 2.18 bc	12.64 ± 2.17 c	24.94 ± 4.55 ab	19.35 ± 0.52 bc	9.89 ± 1.28 c	36.15 ± 4.3 a	7.79 ± 0.96 c
		2	10.82 ± 1.96 a	1.44 ± 0.34 d	6.42 ± 1.81 abc	3.45 ± 0.89 bcd	0.80 ± 0.10 d	8.96 ± 1.46 abc	4.05 ± 0.81 bcd	3.47 ± 0.70 bcd	2.42 ± 0.43 cd	9.49 ± 1.20 ab
		*Xs*	13.58 ± 3.8 ab*	6.53 ± 5.63 c*	8.76 ± 2.90 bc*	8.44 ± 5.72 bc*	6.72 ± 5.63 c*	16.95 ± 11.51 ab	11.70 ± 8.41 bc*	6.66 ± 4.72 c	19.28 ± 18.72 c*	8.64 ± 1.79 bc
	Arbutin	1	425.90 ± 18.2 b	190.24 ± 26.3 cd	75.07 ± 7.16 d	249.48 ± 36.79 c	436.04 ± 62.35 b	641.29 ± 57.02 a	451.31 ± 22.65 b	647.40 ± 24.95 a	630.62 ± 69.51 a	127.36 ± 19.57 d
		2	438.8 ± 44.59 a	96.14 ± 5.98 c	67.34 ± 2.88 c	110.07 ± 17.01 c	102.87 ± 19.99 c	472.48 ± 25.16 a	243.52 ± 34.75 b	246.59 ± 26.15 b	60.63 ± 12.53 c	122.70 ± 21.87 bc
		*Xs*	432.4 ± 31.3 b	291.8 ± 54.2 ed*	71.20 ± 6.46 e	179.8 ± 80.5 d*	269.5 ± 186.1 c*	556.9 ± 126.9 a*	347.4 ± 116.8 c*	447.0 ± 220.7 b*	345.6 ± 300.4 c*	125.03 ± 18.7 ed
	Total phenols	1	850.1 ± 21.6 b	392.88 ± 38.7 cd	309.85 ± 13.21 d	787.21 ± 53.3 b	818.7 ± 88.6 b	1322.92 ± 116 a	541.75 ± 24.97 c	1446.59 ± 59.8 a	848.32 ± 95.18 b	525.96 ± 76.87 c
		2	725.6 ± 39.9 ab	212.7 ± 7.7 cde	197.90 ± 11.11 cde	316.43 ± 57.89 c	159.74 ± 20.1 de	809.6 ± 76.36 a	276.3 ± 34.7 cd	611.79 ± 63.97 b	106.78 ± 16.67 e	237.58 ± 32.57 cd
		*Xs*	787.8 ± 74.02 b*	302.77 ± 103.5 ef*	253.87 ± 62.27 f*	551.8 ± 310.73 c*	489.2 ± 368.2 cd*	1066.28 ± 62.27 a*	409 ± 47.9 de*	1029 ± 460.6 a*	477.55 ± 310.73 cd*	381.8 ± 166.54 de*
PULP	Chlorogenic acid	1	1.57 ± 0.08 ef	3.61 ± 0.75 de	1.63 ± 0.32 ef	14.94 ± 0.9 a	4.42 ± 0.4 d	9.36 ± 1.25 c	1.43 ± 0.1 ef	11.09 ± 0.8 bc	0.46 ± 0.01 f	13.76 ± 0.34 ab
		2	1.99 ± 0.11 c	5.68 ± 1.01 b	1.72 ± 0.23 c	9.69 ± 2.81 a	0.44 ± 0.18 c	6.00 ± 1.56 b	0.10 ± 0.00 c	6.70 ± 1.14 ab	0.45 ± 0.08 c	0.67 ± 0.18 c
		*Xs*	1.78 ± 0.24 d*	4.64 ± 1.70 c	1.68 ± 0.45 d	12.32 ± 3.42 a*	2.43 ± 2.12 d*	7.68 ± 2.42 b	0.77 ± 0.62 d*	8.89 ± 2.56 b*	0.45 ± 0.05 d	7.22 ± 6.71 b*
	Catechin	1	0.83 ± 0.03 b	0.65 ± 0.03 bcd	0.10 ± 0.00 f	1.77 ± 0.07 a	0.4 ± 0.02 def	0.75 ± 0.1 bc	0.21 ± 0.00 ef	1.57 ± 0.06 a	0.46 ± 0.01 cde	0.15 ± 0.00 ef
		2	0.12 ± 0.00 d	0.66 ± 0.06 bc	0.1 ± 0.00 d	0.28 ± 0.05 cd	0.12 ± 0.00 d	1.82 ± 0.28 a	1.16 ± 0.1 b	0.16 ± 0.01 cd	0.19 ± 0.02 cd	0.1 ± 0.00 d
		*Xs*	0.47 ± 0.3 de*	0.58 ± 0.56 cd	0.13 ± 0.03 f*	1.03 ± 0.81 ab*	0.26 ± 0.19 ef	1.29 ± 0.64 a*	0.69 ± 0.52 cd*	0.87 ± 0.77 bc*	0.33 ± 0.20 ef	0.12 ± 0.02 f*
	Epicatechin	1	0.46 ± 0.03 d	0.27 ± 0.00 d	0.7 ± 0.10 d	10.08 ± 0.67 b	0.54 ± 0.01 d	18.78 ± 2.21 a	0.33 ± 0.01 d	4.33 ± 0.8 c	0.31 ± 0.01 d	4.56 ± 0.85 c
		2	0.25 ± 0.00 d	0.17 ± 0.00 e	0.32 ± 0.00 c	0.21 ± 0.00 de	0.24 ± 0.00 d	0.39 ± 0.00 b	0.72 ± 0.04 a	0.21 ± 0.00 de	0.11 ± 0.00 f	0.19 ± 0.00 e
		*Xs*	0.35 ± 0.11 d*	0.22 ± 0.05 d*	0.51 ± 0.22 d*	5.15 ± 4.85 b*	0.39 ± 0.16 d*	9.59 ± 9.25 a*	0.52 ± 0.21 d*	2.27 ± 1.98 c*	0.21 ± 0.10 d*	2.37 ± 2.25 c*
	Arbutin	1	35.4 ± 0.24 bc	21.05 ± 2.18 cd	4.34 ± 0.27 d	20.28 ± 1.14 cd	47.95 ± 5.36 ab	59.83 ± 9.34 a	24.76 ± 0.36 c	51.87 ± 6.98 ab	46.42 ± 8.69 ab	16.95 ± 1.73 cd
		2	64.36 ± 4.85 a	21.45 ± 3.49 bc	6.62 ± 1.12 de	17.11 ± 4.01 bcd	11.62 ± 1.85 cde	66.05 ± 7.02 a	23.43 ± 1.17 b	25.2 ± 3.11 b	18.91 ± 2.77 bc	3.0 ± 0.42 e
		*Xs*	49.9 ± 16.15 b*	21.25 ± 2.61 de	5.48 ± 1.44 f*	18.69 ± 3.16 ef	29.78 ± 20.2 cd*	62.94 ± 12.31 a	24.10 ± 1.06 de	38.5 ± 15.47 c*	32.67 ± 16.13 cd*	9.97 ± 7.72 f*
	Total phenols	1	38.25 ± 0.32 cde	25.58 ± 3.17 ef	6.77 ±1.01 f	47.07± 1.42 cde	53.31 ± 5.6 bc	88.73 ± 9.99 a	26.73 ± 0.5 def	68.86 ± 8.86 ab	47.66 ± 5.73 bcd	35.42 ± 2.52 cde
		2	66.72 ± 4.82 a	27.97 ± 4.0 bc	8.82 ± 1.1 de	27.29 ± 4.46 bc	12.42 ± 1.96 de	74.26 ± 5.96 a	25.42 ± 1.34 bc	32.27 ± 4.01 b	19.66 ± 2.77 cd	3.96 ± 0.30 f
		*Xs*	52.48 ±15.89 b*	26.77 ± 3.48 cd	7.79 ± 1.50 d	37.18 ±11.71 c*	32.87 ± 22.71 c*	81.50 ± 14.88 a	26.07 ± 1.15 cd	50.57 ± 21.1 b*	33.65 ± 16.38 c*	19.69 ± 11.23 d
WHOLE FRUIT	Total phenols	1	888.37 ± 21.9 b	418.46 ± 51.51 cd	316.62 ± 12.2 d	834.28 ± 54.7 b	872.01 ± 97.2 b	1411.7 ± 98.28 a	568.48 ± 25.4 c	1515.5 ± 67.9 a	895.88 ± 78.83 b	561.38 ± 69.37 c
		2	792.28 ± 37.07 a	240.64 ± 5.99 cde	206.72 ± 10.2 de	343.72 ± 32.61 c	172.16 ± 21.9 e	883.91 ± 81.42 a	301.7 ± 35.85 cd	644.07 ± 60.78 b	126.44 ± 19.11 e	241.54 ± 32.79 cde
		*Xs*	840.3 ± 21.93 b*	329.55 ± 102.8 fg*	261.67 ± 61.02 g*	589.0 ± 273.1 c*	522.1 ± 381.7 cd*	1147.8 ± 300.8 a*	435.1 ± 148.7 def*	1079.8 ± 481 a*	511.2 ± 326.7 cde*	401.46 ± 183.4 ef*

Average values ± standard deviation (SD) in rows marked with different letters (a–f) represent statistically significant differences between cultivars; an asterisk indicates statistically significant differences in the same pear cultivar through different years; Tukey’s test, *p* < 0.05; Q-glucoside: quercetin 3-*O*-glucoside; Q-rutinoside: quercetin 3-*O*-rutinoside.

## Data Availability

The data supporting the reported results is available at request from the corresponding author.
